# DADS Regulates EMT and Chemotherapy Resistance by Inhibiting ROR**α**/**β**-Catenin Signaling through PKC**α**-Dependent Phosphorylation in Gastric Cancer

**DOI:** 10.32604/or.2025.068689

**Published:** 2025-11-27

**Authors:** Yizhen Zhang, Juan Li, Huanqing Liu, Hong Xia, Jian Su, Fang Liu, Bo Su, Qi Su

**Affiliations:** 1 Hunan Province Key Laboratory of Cancer Cellular and Molecular Pathology, Cancer Research Institute, University of South China, Hengyang, 421001, China; 2 Department of Pathology, The Affiliated Hospital, Jinggangshan University, Ji’an, 343000, China; 3 Department of Geriatric Medicine, Loudi Central Hospital, Loudi, 417000, China; 4 Department of Oncology, Changsha County People’s Hospital, Changsha, 410100, China; 5 Hunan Clinical Research Center for Gastric Cancer Prevention and Treatment, Second Affiliated Hospital, University of South China, Hengyang, 421001, China; 6 Key Laboratory for Pharmacoproteomics of Hunan Provincial University, Institute of Pharmacy and Pharmacology, University of South China, Hengyang, 421001, China

**Keywords:** Diallyl disulfide, retinoid-related orphan receptor alpha (RORα)/β-catenin signaling, gastric cancer cells, epithelial-mesenchymal transition, drug resistance

## Abstract

**Objectives:**

Gastric cancer (GC) is often associated with high invasiveness, epithelial-mesenchymal transition (EMT), and resistance to 5-fluorouracil (5-FU), highlighting the need for novel therapeutic targets. This study explored whether diallyl disulfide (DADS) upregulates retinoic acid-related orphan receptor alpha (RORα) to weaken the protein kinase C alpha (PKCα)/RORα-mediated RORα/β-catenin pathway, thereby inhibiting GC cell invasion, epithelial-mesenchymal transition (EMT), and enhancing 5-FU sensitivity.

**Methods:**

Human GC cell lines MGC-803 and SGC7901 were treated with DADS, RORα agonist SR1078/antagonist T0901317, and PKCα agonist TPA/antagonist GO6976. Cell proliferation (MTT), migration (scratch assay), invasion (Transwell), protein expression (Western blot), protein interactions (coimmunoprecipitation), and localization (immunofluorescence) were detected. Apoptosis and 5-FU sensitivity-related proteins were examined. Experiments were triplicated; statistics used *t*-test/ANOVA (*p* < 0.05).

**Results:**

DADS/SR1078 inhibited GC cell proliferation/migration/invasion, upregulated RORα/E-cadherin, downregulated nuclear β-catenin/TGF-β1/Rac1/Vimentin, and weakened EMT (reversed by T0901317). DADS/TPA upregulated RORα/p-RORα/PKCα/p-PKCα, promoted PKCα-RORα binding, and downregulated RORα/β-catenin target genes (counteracted by GO6976). DADS upregulated caspase-3 and downregulated Bcl-2/P-gp/XIAP via RORα, promoting apoptosis and 5-FU sensitivity.

**Conclusion:**

DADS inhibits GC progression and enhances 5-FU sensitivity by PKCα/RORα-mediated downregulation of RORα/β-catenin signaling, paralleling SR1078/TPA effects. It may act as a novel RORα agonist for GC therapy.

## 1 Introduction

Gastric cancer (GC) is among the most prevalent malignant neoplasms worldwide [1]. Based on the 2022 global cancer statistics, the new cases of gastric cancer were 968,350 (4.9%) and the deaths of gastric cancer were 659,853 (6.8%), ranking fifth, respectively [1]. Due to the large population in China, about 44% of the global GC (478,508 cases) occurred in China, and the incidence and mortality rates are the third, respectively [2]. Since most patients have already developed invasion and metastasis during treatment, the effect of surgery and chemotherapy is poor, and there is drug resistance, and the 5-year survival rate is less than 10% [2]. Therefore, it is a key problem to study the invasion and metastasis mechanisms of GC and search for therapeutic drugs and targets.

Retinoid-related orphan receptor α (RORα) plays a pivotal role in signal integration and the regulation of gene expression through interactions with various co-regulatory proteins across multiple tumors [3]. Downregulation and reduced activation of RORα have been implicated in tumor development and progression, establishing it as a recognized tumor suppressor [4]. It is well known that the synthetic RORα agonist SR1078 can upregulate and activate RORα, thereby enhancing and stabilizing P53 gene expression and promoting cell apoptosis [4]. In breast cancer cells, the expression of RORα significantly inhibits Snail transcription by binding RORα to ROREs in the promoter region of the Snai1 gene. The results revealed a novel function of RORα in suppressing EMT and identified Snail as a direct target of RORα [5]. Studies have confirmed that downregulation of RORα is a clinical feature of liver cancer. RORα knockdown facilitates EMT via the Wnt/β-catenin pathway and enhances the proliferation, invasion, and metastasis of liver cancer [6]. Studies have shown that RORα deletion can promote the proliferation of GC cells, while SR1078 can reverse this phenomenon and enhance the sensitivity to fluorouracil [7].

Numerous studies have identified diallyl disulfide (DADS) as a key organosulfur compound in garlic. Research has demonstrated that DADS possesses potent anti-tumor effects across multiple cancer cell types, including gastric cancer. The mechanisms of anticancer action of DADS inhibit EMT, invasion, and migration [8,9]. Our previous proteomic analysis showed that DADS can significantly upregulate the expression of RORα in human gastric cancer MGC803 cells [10]. Protein kinase C alpha (PKCα) was shown to phosphorylate RORα and then inhibit β-catenin co-transcription activity [11]. The PKCα agonist TPA can increase the phosphorylation level of RORα [12]. However, whether DADS inhibits invasion and EMT, and drug resistance in human gastric cancer cells through PKCα-dependent phosphorylation mediated reduction of RORα/β-catenin signaling remains to be further investigated. In this study, we investigated the effects of DADS on the proliferation, invasion, migration, EMT, and drug sensitivity of gastric cancer cells, and confirmed that DADS has the same effects as agonist SR1078 and TPA.

## 2 Materials and Methods

### 2.1 Reagents and Antibodies

DADS was purchased from MilliporeSigma (Burlington, MA, USA). SR1078 was purchased from MedChem Express (Monmouth Junction, NJ, USA). T0901317 was purchased from Shanghai Baili Biology Ltd. (Shanghai, China). The primary antibodies β-catenin (sc-1496, 1:1000), PKCα (sc-8393, 1:500), p-PKCα (sc-12356, 1:500), p-β-catenin (sc-101650, 1:1000), Axin (sc-14029, 1:800), and β-actin (sc-8432, 1:2000) were purchased from Santa Cruz Biotechnology (Dallas, TX, USA). RORα (ab60134, 1:1000), TGF-β1 (ab92486, 1:500), Rac1 (ab33186, 1:800), Vimentin (ab92547, 1:1000), E-cadherin (ab40772, 1:1000), Bcl-2 (ab692, 1:1000), caspase-3 (ab13847, 1:1000), P-gp (ab261736, 1:1000), XIAP (ab227196, 1:1000), and horseradish peroxidase (HRP)-conjugated secondary antibodies (ab6717, 1:2000) were obtained from Abcam (Cambridge, UK). c-Jun (P05412, 1:500), c-Myc (P01106, 1:500), and Cyclin D1 (P24385, 1:500) were purchased from Epitomics (Burlingame, CA, USA). The primary antibody against p-β-catenin from Cell Signaling Technology (Danvers, MA, USA) was used at a 1:1000 dilution. The p-RORα Serine 35 antibody (WG-00021) was provided by Shanghai ImmunoGen Biological Technology (Shanghai, China) and used at a 1:500 dilution. TPA and Go6976 were obtained from Sigma-Aldrich Corporation (Burlington, MA, USA).

### 2.2 Cell Culture and Treatment

The gastric cancer cell lines MGC-803 and SGC7901 were obtained from the American Type Culture Collection (ATCC; Manassas, VA, USA). Cell line authentication was performed by Short Tandem Repeat (STR) profile analysis at Beijing Microread Genetics Co., Ltd. (Beijing, China). No mycoplasma contamination was detected throughout the experiments. Both cell lines were cultured in RPMI-1640 medium (Cat#11875119, Thermo Fisher Scientific, Waltham, MA, USA) supplemented with 10% fetal bovine serum (FBS; Cat#10099141, Gibco, Thermo Fisher Scientific, Waltham, MA, USA), 100 U/mL penicillin and 100 µg/mL streptomycin (Cat#15140122, Gibco, Thermo Fisher Scientific, Waltham, MA, USA). Cells were maintained at 37°C in a humidified atmosphere containing 5% CO_2_. Logarithmically growing SGC7901 cells were seeded into 96-well plates at ~5 × 10^3^ cells/well. 5-FU (Cat# 51-21-8, Sigma-Aldrich, Saint Louis, MO, USA) was added to reach final concentrations of 1.25, 2.5, 5, 10, and 20 mg/L; the control group received equal-volume serum-free medium without 5-FU.

### 2.3 Cell Proliferation Analysis

MGC-803 and SGC7901 cells were seeded and allowed to adhere for 8 h, then the medium was replaced with fresh RPMI-1640 (Cat#11875119, Thermo Fisher Scientific). MTT solution (5 mg/mL) was added at 10% of the culture volume and incubated for 4 h at 37°C. After removing the medium, formazan crystals were dissolved in DMSO, and absorbance was measured at 570 nm using a microplate reader (Synergy HTX, BioTek Instruments, Inc., Winooski, VT, USA).

### 2.4 Cell Scratch Test

1 mL of MGC-803 cells (1 × 10^6^/mL) suspension was injected into 6-well plates, with 3 parallel samples in each group. The cells were cultured until monolayers were formed. The cells were scratched on the cell plate with a 10 µL Tip, washed in serum-free medium 3 times, and fresh serum-free medium was added. The relative distance of the scratch area was measured under the microscope (Olympus IX73, Olympus Corporation, Tokyo, Japan).

### 2.5 Migration Assay

The MGC-803 cells were digested by 0.25% trypsin, the supernatant was removed after centrifugation, and serum-free medium was added to make a 2 × 10^5^ cell suspension. 200 μL was absorbed and added to the upper part of the Transwell chamber (Cat#3422, Corning Inc., Corning, NY, USA), serum-free DMEM medium was added to the lower chamber, and cultured for 12 h. The Transwell chamber was removed, the medium in the lower chamber was sucked out, and the pores were cleaned three times with PBS (pH 7.4, 1× PBS solution; Gibco, Thermo Fisher Scientific, Waltham, MA, USA). Fixed with 4% paraformaldehyde, cleaned the chamber 3 times with PBS, stained with 0.1% crystal violet, wiped off the upper cells, and washed with PBS 3 times. The bottom membrane of the chamber was cut and placed on the carrier plate, sealed with glycerin, and counted.

### 2.6 Invasion Experiment

The mixture of Matrigel and RPMI1640 medium was spread on the upper layer of the Transwell cell, and the MGC-803 cells (1 × 10^6^/mL) were placed in a 24-well plate. 24 h later, the cell was fixed with 4% paraformaldehyde and stained with 0.1% crystal violet. Images were captured under an inverted microscope (Nikon Eclipse Ti, Nikon Corporation, Tokyo, Japan), and the number of cells was counted; the average count was used as the experimental result.

### 2.7 Western Blot

The nuclear protein of MGC-803 and SGC7901 cells was extracted according to the nucleoprotein extraction kit (Cat# HR0041, Biorab, Beijing, China). The protein was quantified by the BCA method (Cat# P0010, Beyotime Biotechnology, Shanghai, China). The sample of 10% separation glue was added at 50 μg protein per well for SDS-PAGE electrophoresis. The protein in the gel was transferred to a PVDF membrane at 4°C and constant pressure of 100 V. 5% skim milk was sealed at 4°C overnight. The primary antibody (as described above) was incubated overnight at 4°C, the secondary at 37°C for 2 h, followed by exposure, development, and fixation in the dark.

### 2.8 Coimmunoprecipitation

Nuclear extracts were isolated following the protocol provided by the manufacturer using the NE-PER^®^ Kit (Pierce, Thermo Fisher Scientific, Rockford, IL, USA). Immunoprecipitation was performed utilizing Protein A/G agarose beads (Cat# sc-2003, Santa Cruz Biotechnology, Dallas, TX, USA), and the obtained pellets were subjected to three washes with 1X RIPA buffer (pH 7.4). Subsequently, the immunoprecipitated proteins were examined by Western blot analysis.

### 2.9 Immunofluorescence Assays

MGC-803 and SGC7901 cells were fixed with 4% paraformaldehyde, permeabilized with 0.5% Triton X-100 in PBS, and blocked with goat serum (Cat# SL038, Solarbio, Beijing, China). The cells were then incubated with primary antibody (as described above) at 37°C for 1 h, followed by FITC-conjugated secondary IgG antibody for 1 h at 37°C. The samples were incubated with DAPI (Cat# D1306, Invitrogen, Carlsbad, CA, USA) for 5 min at room temperature.

### 2.10 Flow Cytometry Analysis

Cells from SGC-7901, VCR, and T0901317 groups were stained with Annexin V-FITC and propidium iodide using the Annexin V-FITC Apoptosis Detection Kit (CA1020, BD Biosciences, Franklin Lakes, NJ, USA) according to the manufacturer’s instructions. After staining, cells were analyzed by flow cytometry (BD FACSCanto II, BD Biosciences) to determine apoptosis rates. Data were collected from at least 1 × 10^4^ cells per sample and analyzed with FlowJo software (Version 10, Tree Star Inc., Ashland, OR, USA).

### 2.11 Statistical Analysis

All data are expressed as mean ± standard deviation (SD) from three independent experiments. Differences in expression among groups were analyzed using Student’s *t*-test and one-way ANOVA. The *p*-values < 0.05 were regarded as indicative of statistical significance. All statistical analyses were performed using SPSS version 13.0 software (IBM Corp., Armonk, NY, USA).

## 3 Results

### 3.1 Effects of DADS and RORα Agonist SR1078 on Proliferation, Migration, and Invasion in Human Gastric Cancer Cells

**Figure 1:**
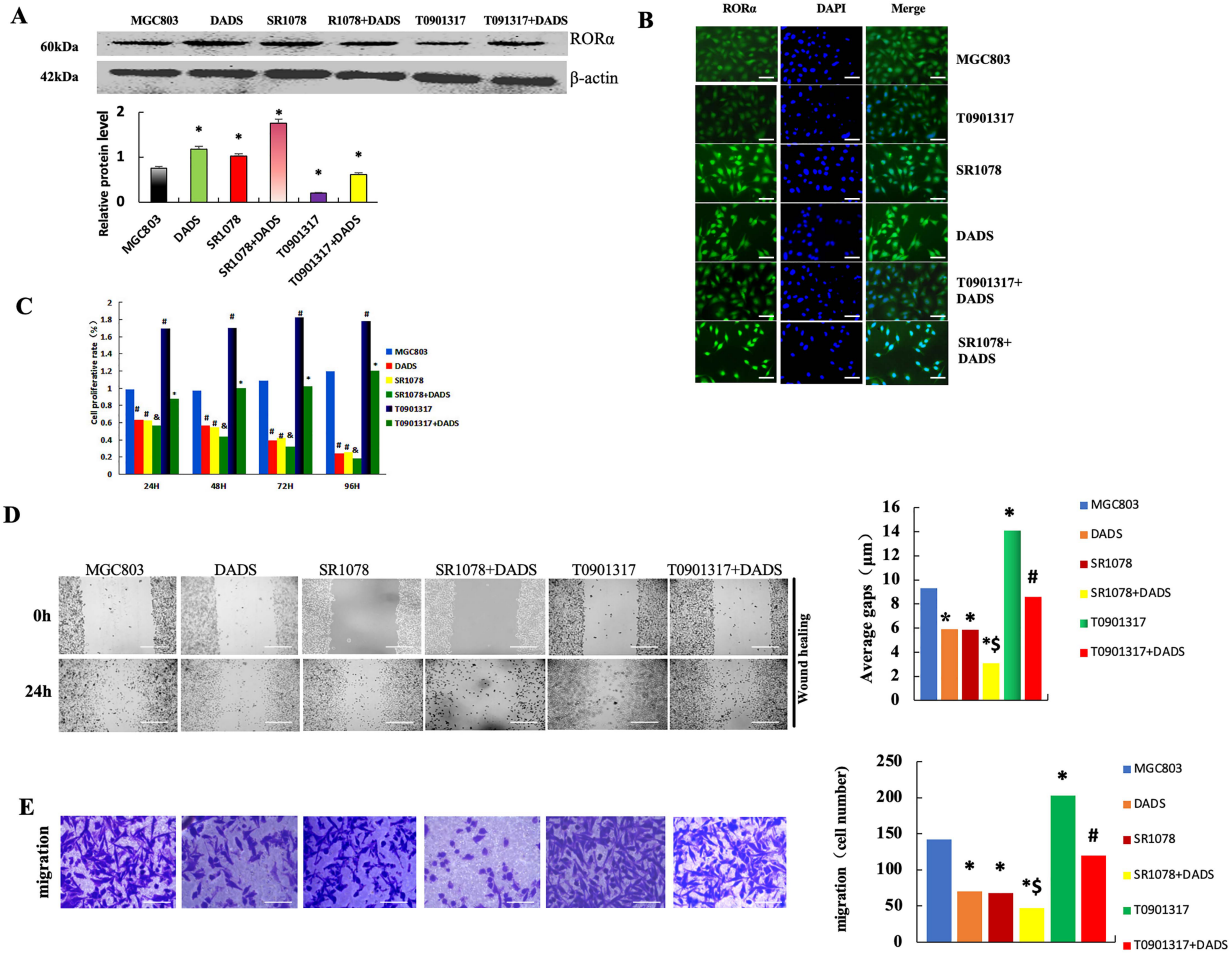

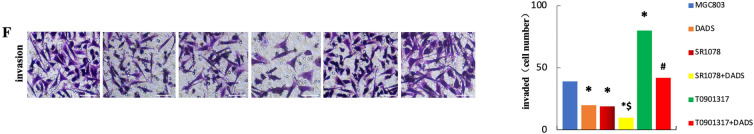
Effects of DADS and RORα agonist SR1078 on proliferation, migration, and invasion in human gastric cancer cells. (**A**) Expression of RORα protein (*p < 0.05). (**B**) Immunofluorescence was consistent with that of the Western blot (Scale bar = 20 μm). (**C**) DADS and SR1078 inhibit in a time-dependent manner the proliferation in MGC803 cells (^#^*p* < 0.05 represents significant differences compared with the MGC803; ^&^*p* < 0.05 represents significant differences compared with the SR1078; *p < 0.05 represents significant differences compared with the T0901317) (**D**) Scratch test showed that DADS and SR1078 inhibited migration (*p < 0.05), and DADS enhanced SR1078 (^$^*p* < 0.05), and the T0901317+DADS group decreased compared with the T0901317 group (^#^*p* < 0.05) (×4) (Scale bar = 200 μm). (**E**) Migration assay shows that DADS and SR1078 inhibit MGC803 cells' migration (*p < 0.05), and DADS can enhance the SR1078 effect (^$^*p* < 0.05). And the T0901317+DADS group decreased compared with the T0901317 group (^#^*p* < 0.05) (×20) (Scale bar = 40 μm). (**F**) Invasion experiment showed that DADS and SR1078 can inhibit the invasion of MGC803 cells (*p < 0.05), and DADS can enhance the effect of SR1078 (^$^*p* < 0.05). And the T0901317+DADS group decreased compared with the T0901317 group (^#^*p* < 0.05) (×40) (Scale bar = 20 μm)

To demonstrate that DADS is an RORα agonist, we used SR1078 as a positive control and antagonist T090131 as a negative control to investigate the effects of DADS and SR1078 in GC cell migration, invasion, and EMT. As shown in Fig. 1A, RORα protein expression was increased in the DADS and SR1078 groups (*p* < 0.05), with DADS further enhancing the effect of SR1078 (*p* < 0.05). Conversely, RORα expression was downregulated in the T0901317 group (*p* < 0.05), while the T0901317+DADS group exhibited higher RORα expression than the T0901317 group alone (*p* < 0.05). These results indicate that both DADS and SR1078 upregulate RORα expression with comparable efficacy, suggesting that DADS acts as a RORα agonist. Immunofluorescence findings were consistent with these results (Fig. 1B). As depicted in Fig. 1C, MGC803 cell proliferation in the DADS and SR1078 groups significantly decreased in a time-dependent manner (*p* < 0.05), with no significant difference observed between these two groups (*p* > 0.05). Although DADS exhibited similar inhibitory effects on proliferation as SR1078, the proliferation rate in the SR1078+DADS group was lower (*p* < 0.05). In contrast, the T0901317 group showed a significant increase in proliferation (*p* < 0.05), which was attenuated in the T0901317+DADS group (*p* < 0.05; Fig. 1C). These findings demonstrate that DADS mimics the effects of SR1078 and can potentiate its activity.

The scratch assay demonstrated comparable scratch distances across all groups at 0 h. After 24 h, the scratch distances in the DADS and SR1078 groups decreased significantly (*p* < 0.05), with the SR1078+DADS group exhibiting a greater reduction than either the DADS or SR1078 groups alone (*p* < 0.05). Similarly, the T0901317+DADS group showed a significant decrease relative to the T0901317 group (*p* < 0.05) (Fig. 1D). In the migration assay, the number of migrated cells was significantly lower in the SR1078 and DADS groups compared to the MGC803, T0901317, and T0901317+DADS groups (*p* < 0.05). Moreover, the SR1078+DADS group had fewer migrated cells than the SR1078 or DADS groups alone (*p* < 0.05). The T0901317 group exhibited a significantly higher number of migrated cells than the MGC803 group (*p* < 0.05), while the T0901317+DADS group showed a significant reduction compared to the T0901317 group (*p* < 0.05) (Fig. 1E). These results indicate that DADS and SR1078 effectively inhibit gastric cancer cell migration, with DADS enhancing the inhibitory effect of SR1078. The invasion assay revealed a significant decrease in invaded cells in the DADS and SR1078 groups relative to the MGC803, T0901317, and T0901317+DADS groups (*p* < 0.05). The SR1078+DADS group demonstrated a further reduction compared to the SR1078 group alone (*p* < 0.05). Additionally, the T0901317+DADS group had significantly fewer invaded cells than both the MGC803 and T0901317 groups (*p* < 0.05) (Fig. 1F). These findings suggest that DADS and SR1078 inhibit gastric cancer cell invasion and that DADS enhances the inhibitory effect of SR1078.

### 3.2 Effect of DADS and SR1078 on RORα/β-Catenin Signaling

#### 3.2.1 DADS and SR1078 Promote the Binding of RORα Protein to *β*-Catenin Protein and Downregulate Nucleus-*β*-Catenin Expression

**Figure 2:**
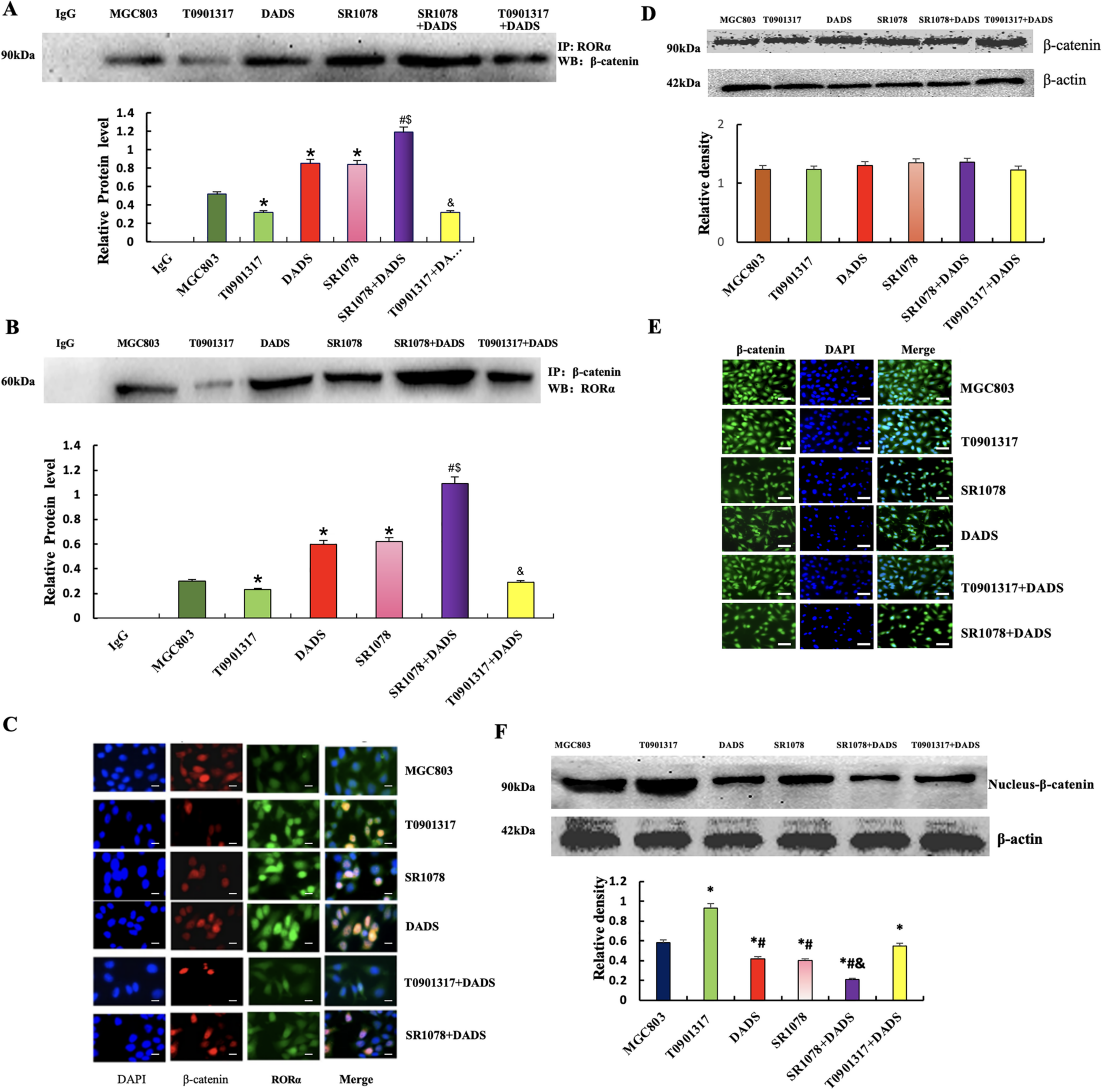
Effect of DADS and SR1078 on RORα/β-catenin signal. (**A** and **B**) Co-immunoprecipitation showed that the combination of RORα and β-catenin (*p < 0.05 represents significant differences compared with the MGC803; ^$^*p* < 0.05 represents significant differences compared with the DADS; ^#^*p* < 0.05 represents significant differences compared with the SR1078; ^&^*p* < 0.05 represents significant differences compared with the T0901317.). (**C**) Dual standard immunofluorescence confirmed that DADS and SR1078 promote the binding of RORα to β-catenin (Scale bar = 20 μm). (**D** and **E**) Western blot and Immunofluorescence showed that β-catenin was expressed with no significant difference in all groups (Scale bar = 20 μm). (**F**) The expression of nuclear-β-catenin protein was down-regulated in the SR1078 group and DADS group compared to MGC803, T0901317, and T0901317+DADS groups (^*#^*p* < 0.05), and SR1078+DADS compared to SR1078 and DADS (^&^*p* < 0.05)

Co-immunoprecipitation showed that Total protein was precipitated with β-catenin or RORα antibody, respectively. The binding of RORα to β-catenin in the DADS and SR1078 groups was significantly increased (*p* < 0.05). Furthermore, the SR1078+DADS group exhibited significantly higher binding levels than either the DADS or SR1078 groups alone (*p* < 0.05). Conversely, the T0901317 group showed a significant decrease in RORα and β-catenin binding (*p* < 0.05), while the T0901317+DADS group demonstrated a significant increase (*p* < 0.05) (Fig. 2A,B). Dual-label immunofluorescence confirmed enhanced RORα and β-catenin binding in the DADS and SR1078 groups, consistent with the immunoprecipitation results (Fig. 2C). These findings indicate that DADS and SR1078 promote the interaction between RORα and β-catenin, with DADS enhancing the effect of SR1078.

Western blot analysis revealed a significant reduction in nuclear β-catenin levels in the DADS and SR1078 groups (*p* < 0.05), with comparable effects observed between the two treatments. In contrast, nuclear β-catenin expression was elevated in the T0901317 group (*p* < 0.05) but significantly decreased in the T0901317+DADS group (*p* < 0.05). These findings indicate that DADS mimics the downregulatory effect of SR1078 on nuclear β-catenin and can enhance SR1078’s effect. Immunofluorescence analysis demonstrated β-catenin presence in both the cytoplasm and nucleus, with no significant differences in staining intensity across all groups (Fig. 2D–F).

#### 3.2.2 Effect of DADS and SR1078 on EMT in MGC803 Cells

**Figure 3:**
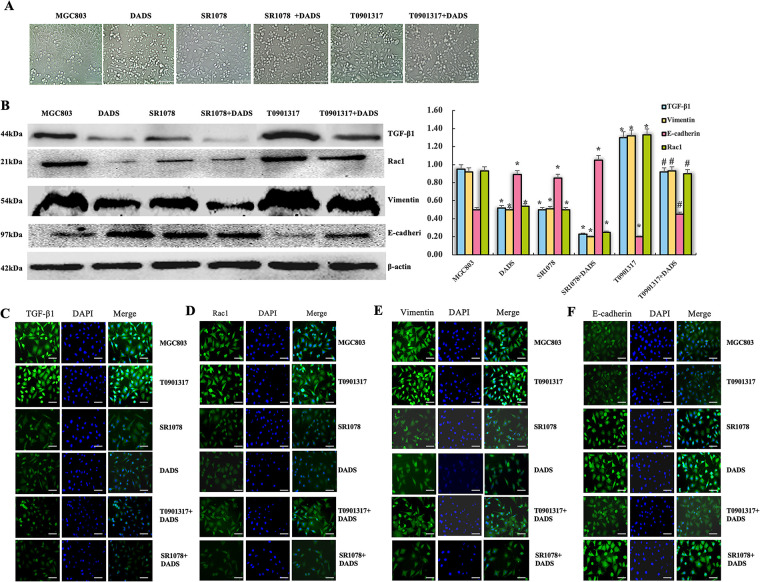
Effect of DADS and SR1078 on EMT in MGC803 cells. (**A**) The cells of the DADS group and the SR1078 group showed atypia decreased (Scale bar = 20 μm). (**B**) DADS group and SR1078 group down-regulated TGF-β1, Rac1, and Vimentin, but up-regulated E-cadherin, and DADS can enhance the SR1078 effect. The T0901317+DADS group compared to the T0901317 group down-regulated or up-regulated (^*#^*p* < 0.05). (**C**–**F**) The immunofluorescence assay results were consistent with those of the Western blot (Scale bar = 20 μm)

Phase contrast microscopy showed that MGC803 cells showed EMT morphology of long spindle cells with obvious polymorphism. After DADS treatment with SR1078, the long spindle cells decreased, some cells became round, and the atypia decreased, while the SR1078+DADS cells were basically round (Fig. 3A). Western blot showed that the expressions of TGF-β1, Rac1, and Vimentin in the DADS group and SR1078 group were significantly down-regulated (*p* < 0.05), while the expression of E-cadherin was up-regulated (*p* < 0.05). The SR1078+DADS group was more significant than the DADS group and the SR1078 group (Fig. 3B). Immunofluorescence showed that TGF-β1, Rac1, and Vimentin protein-positive signal decreased in the DADS group and SR1078 group, and E-cadherin protein was enhanced. The effect of the SR1078+DADS group was more obvious (Fig. 3C–F).

### 3.3 PKCα/RORα Mediated DADS Antagonizing RORα/β-Catenin Signaling

#### 3.3.1 DADS Up-Regulated PKCα and Phosphorylated PKCα

**Figure 4:**
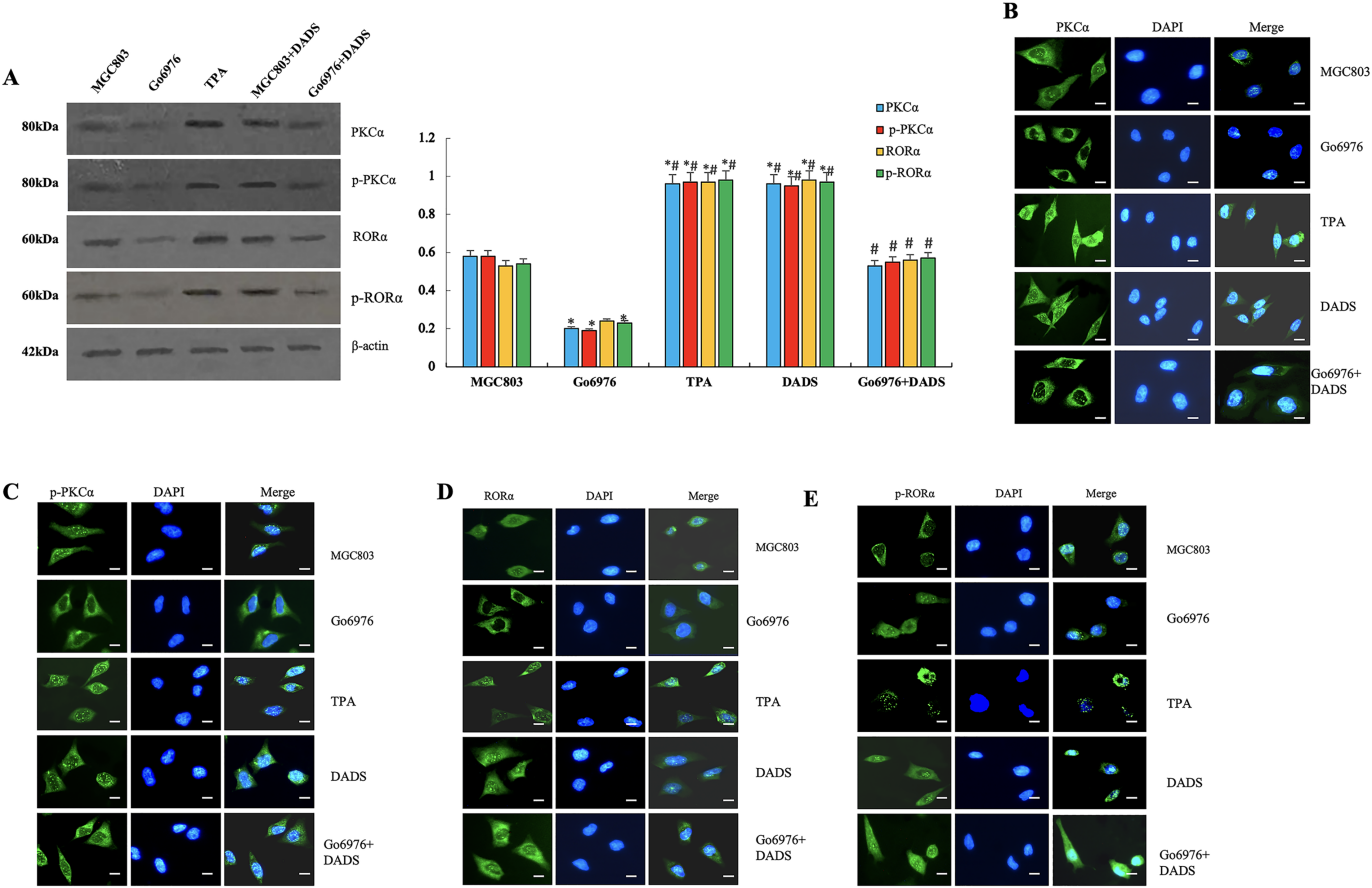
PKCα/RORα mediated DADS antagonizing RORα/β-catenin signaling. (**A**) The expressions of PKCα and p-PKCα were significantly upregulated in the DADS group and the TPA group compared to the MGC803 and Go6976 groups (*p < 0.05). However, in the Go6976 group, the expressions of PKCα and p-PKCα were downregulated (^#^*p* < 0.05), and DADS could weaken the effect of Go6976 (^#^*p* < 0.05). The expressions of RORα and p-RORα were upregulated in the DADS and TPA treatment groups (*p < 0.05), while they were lower in the Go6976 group compared to the MGC803 group (*p < 0.05), and DADS could weaken the effect of Go6976 (^#^*p* < 0.05). (**B**–**E**) The results of immunofluorescence detection were consistent with Western blot (×40). Data are represented as mean ± SD for three independent experiments (Scale bar = 20 μm)

Western blot analysis revealed a significant increase in PKCα and phosphorylated PKCα (p-PKCα) levels in MGC803 cells treated with DADS (*p* < 0.05). Similarly, PKCα and p-PKCα were markedly upregulated in the TPA-treated group (*p* < 0.05), while both proteins were significantly downregulated in the Go6976 group (*p* < 0.05). Notably, the DADS+Go6976 group exhibited a significant elevation in PKCα and p-PKCα levels (*p* < 0.05) (Fig. 4A). The results of immunofluorescence and immunoprecipitation were consistent. It is indicated that DADS can up-regulate and activate PKCα, acting as the PKCα agonist TPA.

#### 3.3.2 DADS Upregulates ROR*α* Protein and Activates ROR*α*

Western blot showed that the expressions of RORα and p-RORα in TPA and DADS groups were significantly upregulated (*p* < 0.05). The expressions of RORα and p-RORα were significantly down-regulated in the Go6976 group (*p* < 0.05), and increased in the Go6976+DADS group than in the Go6976 group (*p* < 0.05) (Fig. 4A). The results of immunofluorescence and Western blot were consistent (Fig. 4B–E). The results showed that DADS can promote RORα phosphorylation by activating PKCα.

#### 3.3.3 DADS Promotes the Binding of PKC*α* to ROR*α*

**Figure 5:**
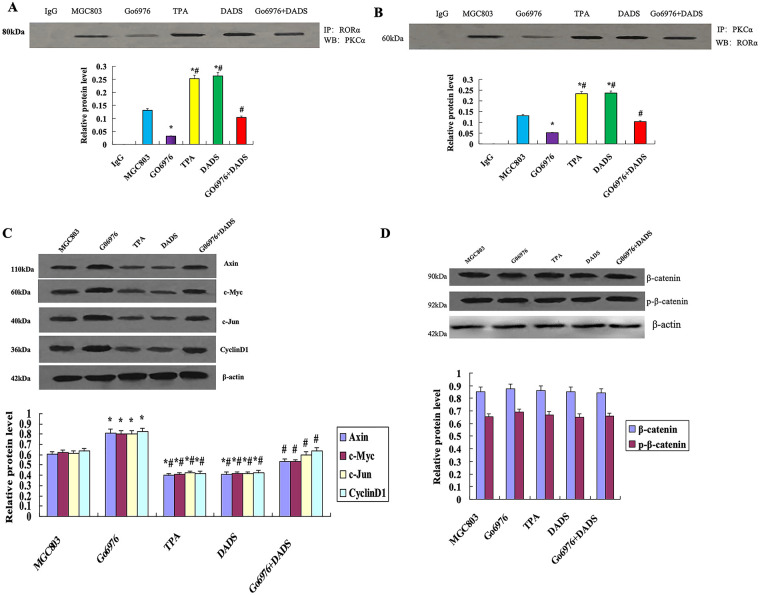
DADS promotes the binding of PKCα to RORα. (**A**) Co-immunoprecipitation RORα showed that the binding of PKCα to RORα increased in the DADS group and TPA group (*p < 0.05). The binding of PKCα and RORα in GO6976 group compared to MGC803 group was down-regulated (*p < 0.05), and DADS can reduce the GO6976 effect (^#^*p* < 0.05). (**B**) Co-immunoprecipitation showed that the binding of RORα to PKCα increased in the DADS group and TPA group than in MGC803 group and GO6976 group (*p < 0.05), and DADS can reduce the Go6976 effect (^#^*p* < 0.05), but the Go6976 group showed down-regulation compared to the MGC803 group (*p < 0.05). (**C**) TPA and DADS down-regulated the expression of Axin, c-Jun, Cyclin1, and c-Myc of RORα/β-catenin signaling (*p < 0.05), and DADS can reduce the Go6976 effect (^#^*p* < 0.05), but the Go6976 group compared to the MGC803 group down-regulated (*p < 0.05). (**D**) DADS and TPA did not affect the expression of β-catenin and P-β-catenin. Data are represented as mean ± SD for three independent experiments

Co-immunoprecipitation of RORα showed that the combination of PKCα and RORα in the DADS group and TPA group was increased compared with that in the untreated group and GO6976 group (*p* < 0.05). The binding of PKCα and RORα in the GO6976 group was down-regulated (*p* < 0.05), while the binding of PKCα and RORα in GO6976+DADS group was up-regulated (*p* < 0.05) (Fig. 5A). The results showed that DADS can promote the binding of PKCα and RORα.

#### 3.3.4 DADS Promotes the Binding of ROR*α* to PKC*α*

Co-immunoprecipitation of PKCα showed that the binding of RORα to PKCα by DADS and TPA was significantly up-regulated (*p* < 0.05), and the expression of RORα was increased in the GO6976+DADS group (*p* < 0.05) (Fig. 5B). The results showed that RORα specifically binds to PKCα, suggesting DADS can promote the binding of RORα to PKCα.

#### 3.3.5 Effect of DADS on the Protein Expression Associated with ROR*α*/β-Catenin Signaling

Western blot showed that the expressions of Axin, c-Jun, Cyclin1, and c-Myc were significantly down-regulated by TPA and DADS (*p* < 0.05). GO6976 could up-regulate the expression of Axin, c-Jun, Cyclin1, and c-Myc (*p* < 0.05) (Fig. 5C). DADS and TPA did not affect the expression of β-catenin and p-β-catenin (*p* > 0.05) (Fig. 5D). These results suggest that DADS may inhibit RORα/β-catenin signaling target genes by activating PKCα.

### 3.4 DADS Up-Regulation of RORα Enhances the Chemotherapy Sensitivity of Human Gastric Cancer Cells to 5-FU

#### 3.4.1 ROR*α* Low Expression in SGC7901 Cells

**Figure 6:**
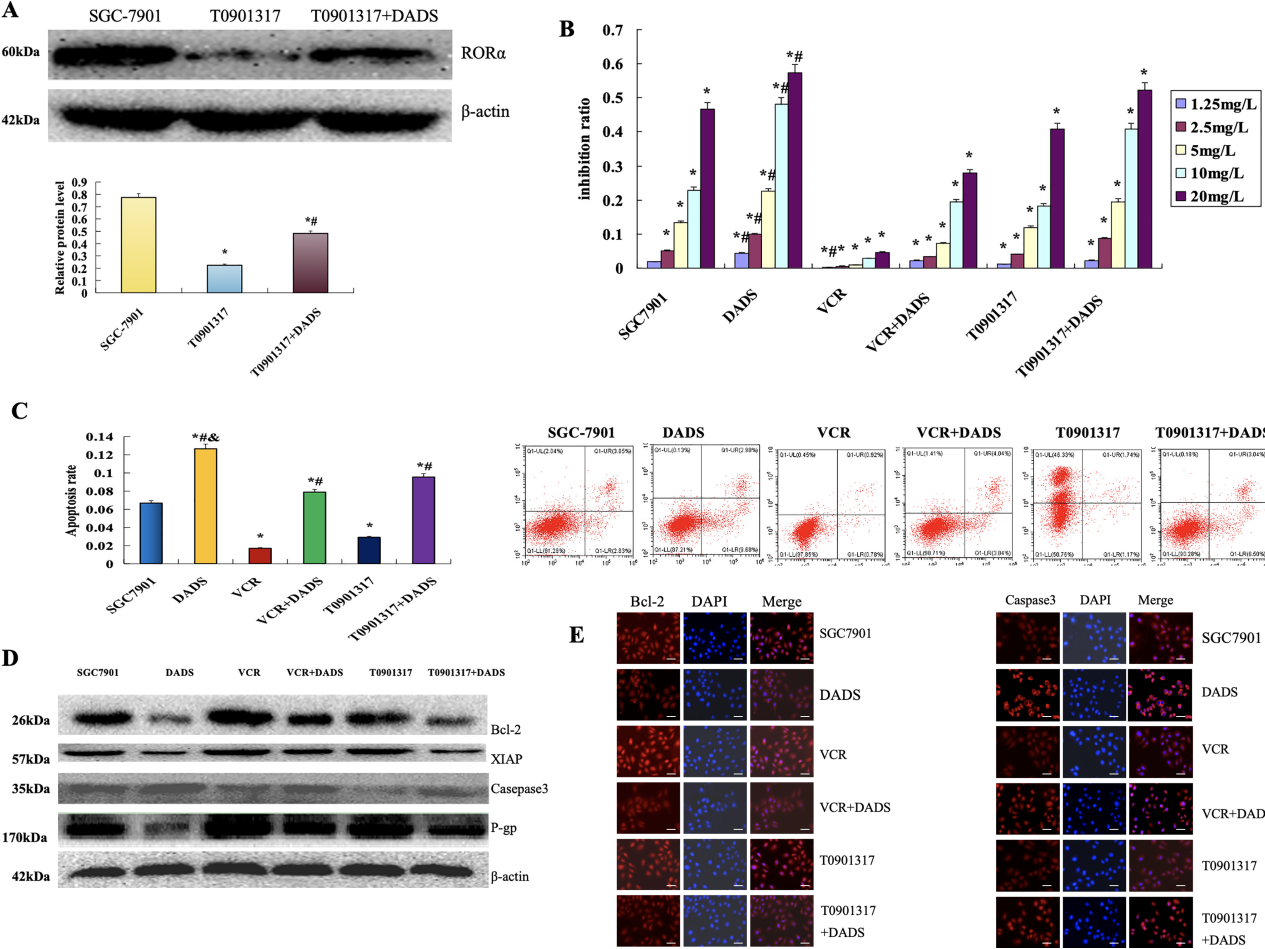

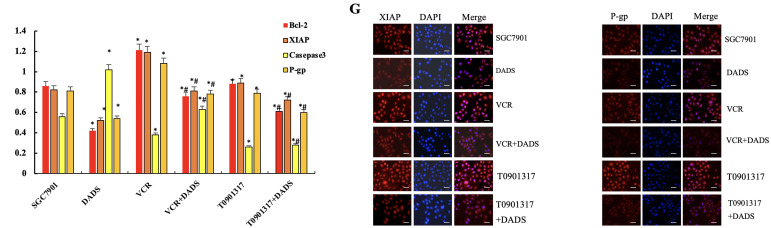
DADS upregulation of RORα enhances the chemotherapy sensitivity of SGC-7901 cells to 5-FU. (**A**) The expression of RORα in SGC-7901 cells treated with T0901317 was down-regulated (*p < 0.05), and DADS up-regulated the expression of RORα (^#^*p* < 0.05). (**B**) After treatment with various concentrations of 5-FU, DADS showed a concentration-dependent increase in inhibition (^*#^*p* < 0.05). (**C**) The apoptosis rates of SGC-7901, VCR, and T0901317 groups are reduced. After DADS treatment, the apoptosis rate in every group increased; VCR and T0901317 groups compared to the SGC-7901 group increased (^*#&^*p* < 0.05). (**D**) In SGC7901, VCR, and T0901317 groups, the expressions of Bcl-2, P-gp, and XIAP were upregulated (*p < 0.05), while the expression of Caspase3 was downregulated (^#^*p* < 0.05). After DADS treatment, the expressions of Bcl-2, P-gp, and XIAP in each group decreased, while the expression of Caspase-3 increased (^#^*p* < 0.05). (**E**–**H**) The results of immunofluorescence detection were consistent with Western blot (×40). Data are represented as mean ± SD for three independent experiments (Scale bar = 20 μm)

Western blot showed that SGC7901 cells were treated by using the RORα inhibitor T0901317. The expression of RORα in SGC7901 cells was significantly decreased (*p* < 0.05). However, the expression of RORα in SGC7901 cells was significantly decreased after DADS treatment, with SGC7901/T0901317 increased (*p* < 0.05) (Fig. 6A).

#### 3.4.2 Effect of DADS and Downregulation of ROR*α* on the Proliferation in SGC7901 Cells

The MTT results showed that the proliferation inhibition rates of SGC7901 were 1.95%, 5.09%, 13.43%, 22.87%, and 46.63% following treatment with 5-FU at concentrations of 1.25–20 mg/L, respectively. The inhibition rate of the SGC7901 group after DADS treatment was 4.33%, 9.85%, 22.5%, 48.17% and 57.44%, respectively (*p* < 0.05). The proliferation inhibition rates of SGC7901/VCR were 0.18%, 0.59%, 1.01%, 2.82% and 4.69%, respectively (*p* < 0.05). The inhibition rates of SGC7901/VCR treated with DADS were 2.23%, 3.31%, 7.3%, 19.43% and 27.91%, respectively (*p* < 0.05). The proliferation inhibition rate of the SGC7901/T0901317 group was 1.24%, 4.3%, 11.83%, 18.34% and 40.89%, respectively. Compared with the SGC7901 group, the proliferation inhibition rate was significantly decreased (*p* < 0.05). The inhibitory rates of SGC7901/T0901317 group after DADS treatment were 2.24%, 8.7%, 19.54%, 40.83% and 52.34%, respectively (*p* < 0.05) (Fig. 6B). The results suggest that down-regulation of RORα expression can enhance cell proliferation and increase the resistance of cells to 5-FU to a certain extent. DADS can inhibit the cell proliferation of SGC7901, SGC7901/VCR, and SGC7901/T0901317, and reduce the cell resistance to 5-FU to a certain extent (Fig. 6C).

#### 3.4.3 The Effect of DADS and Downregulation of ROR*α* on the Apoptosis of SGC-7901 Cells

Flow cytometry analysis showed that the apoptosis rates of the SGC-7901 group, VCR group, and T0901317 group were 6.8%, 1.7%, and 2.91%, respectively. The apoptosis rates of the VCR group and T0901317 group were lower than those of the SGC-7901 group (*p* < 0.05). After treatment with DADS, the apoptosis rates of the SGC-7901 group, VCR group, and T0901317 group were 12.66%, 7.88%, and 9.54%, respectively, which were higher than those of the untreated groups (*p* < 0.05). This indicates that low expression of RORα can reduce the apoptosis of SGC-7901 cells, while DADS can increase cell apoptosis.

#### 3.4.4 Effects of DADS and Down-Regulated ROR*α* on Apoptosis-Related Protein Expression

The results showed that the expression of Bcl-2, P-gp, and XIAP in SGC7901, SGC7901/VCR, and SGC7901/T0901317 groups was significantly increased (*p* < 0.05), and Caspase3 protein decreased (*p* < 0.05). After DADS treatment, the expressions of Bcl-2, P-gp, and XIAP in SGC7901, SGC7901/VCR, and SGC7901/T0901317 groups were significantly decreased, while the expression of Caspase-3 was significantly upregulated (*p* < 0.05). Results suggest that the resistance-related proteins and apoptosis-inhibitory protein can be upregulated by RORα low expression, and the pro-apoptotic protein by low RORα expression. After being treated with DADS, the expression of drug-resistance-related protein and apoptosis protein was down-regulated, and the expression of pro-apoptotic protein was up-regulated. Low expression of RORα can up-regulate the expression of Bcl-2, P-gp, and XIAP, and down-regulate the expression of Caspase-3, which inhibits apoptosis and decreases chemosensitivity to 5-FU in SGC7901 cells. DADS could downregulate the expression of Bcl-2, P-gp, and XIAP, and upregulate the expression of Caspase3 through upregulating the expression of RORα, increasing apoptosis and chemosensitivity of SGC7901 cells to 5-FU (Fig. 6D). (Fig. 6E–H). The results of immunofluorescence detection were consistent with Western blot.

## 4 Discussion

Expression of RORα has been observed in multiple cancers, including gastric, cervical, pancreatic cancers, leukemia, lymphoma, myeloma, and bladder cancer. Moreover, RORα expression suppresses proliferation, promotes apoptosis, and inhibits invasion in breast cancer cells [3]. Low expression levels of RORα were associated with poor overall survival in gastric cancer patients. RORα expression may be positively correlated, with patients exhibiting high RORα expression having the best prognosis [13]. It is shown that RORα is significantly downregulated in OSCC, correlates with advanced stage and poor prognosis, inhibits tumor cell proliferation, and its reduction decreases p53 expression and activity [14]. RORα levels are higher in glioblastoma than in healthy controls. RORα expression was lower in HGG glioblastoma compared to LGG. Moreover, high expression of RORα can inhibit proliferation, migration, and invasion [15]. RORα inhibited the proliferation and invasion of colorectal cancer cells *in vitro* and *in vivo*, and the growth and metastasis of transplanted tumors in nude mice, while the effect of RORα knockdown was contrary [16].

SR1078 is known to be a synthetic RORα agonist [17], and SR1078 can cause p53 stabilization and induce apoptosis [18,19]. In addition, SR1078 inhibited colorectal cancer cell proliferation, colony formation, and migration, and inhibited transplanted tumor growth in nude mice [16]. SR1078 can upregulate and activate RORα, restore the expression and activation of BMAL1, effectively block MYCN-mediated tumor growth, and enhance the sensitivity of neuroblastoma to chemotherapy [20]. SR1078 treatment of HepG2 cells could increase the expression of ROR target gene, up-regulate p53 protein level and its target gene expression, and increase the proportion of subG1 apoptotic cells [21,22]. SR1078 inhibits ovarian cancer cell proliferation and spheroid formation [23]. Low expression of RORα leads to high circulating tumor cell count and up-regulation of VEGF in GC, while silencing RORα can significantly promote the proliferation, up-regulate N-cadherin and Vimentin, and down-regulate E-cadherin. In addition, Ki-67 and PCNA are upregulated *in vivo*; however, SR1078 reverses these changes. Moreover, RORα expression inversely correlates with G6PD and PFKFB3, and low RORα combined with high G6PD or PFKFB3 predicts poor survival in gastric cancer, indicating RORα as a potential biomarker and therapeutic target [7].

In recent years, the study of RORα as a tumor therapeutic target has become a hot spot, especially the search for agonists or drugs from natural chemicals to promote the expression and activation of RORα, which has broad application prospects in the field of cancer therapy. It has been found that the flavonoid nobiletin (NOB), which exists in citrus fruits, acts as an RORα agonist, inducing tumor cell apoptosis and cell cycle arrest, inhibiting migration and invasion, down-regulating many carcinogenic factors, up-regulating tumor suppressor factors, and increasing chemotherapy sensitivity of cancer cells [24,25]. NOB inhibits cancer cell growth via the Nrf2/PI3K/Akt pathway and induces apoptosis by suppressing the PARP2-SIRT1/AMPK axis, inhibits the anti-angiogenic effects of STAT3 and VEGF, upregulates GSK-3β, and downregulates EMT-related factors, inhibits the Wnt/β-catenin pathway, thereby suppressing the migration, invasion, EMT, and metastasis of cancer cells [26]. NOB activates ROR response elements of ROR binding to IκBα promoter via NOB-ROR axis *in vivo* and *in vitro*, inhibits p65 nuclear translocation via NF-κB pathway, and significantly inhibits the proliferation of breast cancer cells [27]. Moreover, NOB could inhibit Cholangiocarcinoma (CCA) cell proliferation [28]. NOB can inhibit the activity of ERK1/2, causing the cells to be arrested at the G0/G1 phase, downregulate cyclin-D1 and upregulate p21, reduce the expression of Bcl-xL, inducing apoptosis, and inhibit the activities of AKT and downstream mTOR, thereby exerting an anti-triple-negative breast cancer cell effect [29]. NOB can downregulate CXCR4 and MMP-9 as well as the activity of MMP-9 by inhibiting NF-κB and activating MAPKs, thereby reducing the invasion of breast cancer cells [30].

PS VII (Chonglou Saponin VII) is a steroid saponin extracted from the roots and stems of plants in the Liliaceae family. It can inhibit glycolysis and angiogenesis, and induce apoptosis of ovarian cancer cells. In addition, PS VII can bind to RORα, activate RORα, and regulate the downstream ECM1/VEGFR2 axis, inhibit the binding with the FAK/AKT/GSK3β pathway, affect glycolysis and angiogenesis, and improve the efficacy of drug-resistant treatment [31]. PS VII suppresses ovarian cancer cell proliferation and glycolysis-induced apoptosis via the RORC/ACK1 pathway, suggesting its potential as a chemotherapy agent [32]. PS VII induces cervical cancer cell apoptosis by modulating caspases, Bax, and Bcl-2, indicating its therapeutic potential [33].

Many studies have shown that DADS, as the main component of garlic organosulfur compounds, may be an anti-tumor drug [8]. The expression of RORα in oral cancer cells is down-regulated, while SR1078 can up-regulate and activate the expression of RORα, inhibit proliferation, migration, invasion, and metastasis, and induce apoptosis, suggesting that SR1078 may be a potential drug for the treatment of oral cancer [34]. DADS can inhibit Cdc25C/cyclin B1 pathway-specific phosphorylation of Chk1 and induce G2/M phase arrest in GC cells [35]. In this study, DADS and SR1078 can inhibit the proliferation, migration, and invasion of GC cells by up-regulating RORα, and DADS has the effect of SR1078 and can enhance the effect of SR1078, suggesting that DADS has the role of RORα activator. But, the RORα inhibitor T0901317 has the opposite effect (Fig. 1). And, DADS and SR1078 promote the binding of RORα protein to β-catenin protein and downregulate Nucleus-β-catenin expression; moreover, decrease TGF-β1, Rac1, and Vimentin protein, and enhance E-cadherin protein. Meanwhile, the number of fibroblast-like cells decreased, some of the cells were round, the atypia was significantly reduced, EMT was weakened, and these changes were more significant in SR1078+DADS cells; however, the RORα inhibitor T0901317 has the opposite effect (Figs. 2 and 3). It is suggested that DADS can inhibit EMT, and the effect is similar to SR1078.

It was shown that Wnt5a/PKCα-dependent phosphorylation on RORα serine residue 35 is the key to the Wnt/β-catenin pathway of RORα, and has an inhibitory effect on the expression of Wnt/β-catenin target genes [12]. PGE2/PKCα-dependent phosphorylation of RORα can weaken the expression of Wnt target genes in colon cancer cells [36]. The expression of PKCα in CC cells was significantly decreased, and the decrease of PKCα expression could promote the growth and tumorigenicity of CC cells, while the increase of PKCα activity could significantly inhibit the invasion and growth of CC cells and promote cell death. Moreover, PKCα can induce RORα phosphorylation, leading to β-catenin co-transcriptional inhibition and trans inhibition of Wnt/β-catenin target genes [11]. As a corepressor, RORα is responsible for downregulating the expression of Wnt/β-catenin target genes. In the noncanonical Wnt pathway, Wnt5a activates PKCα via phosphorylation, and phosphorylation of RORα by activated PKCα can serve as an intersection between the noncanonical and canonical Wnt pathways. Phosphorylated RORα transrepresses β-catenin in the canonical Wnt/β-catenin pathway and downregulates expression of cyclin D1, Axin, c-Jun, and c-Myc [19].

This study showed that DADS and PKCα agonist TPA can significantly up-regulate PKCα and phosphorylated PKCα, up-regulate and activate RORα, promote the binding of PKCα to RORα or promote the binding of RORα to PKCα, and inhibit the expression of RORα/β-catenin signaling target genes Axin, c-Jun, Cyclin1, and c-Myc. However, the results of the PKCα inhibitor GO6976 were reversed in CC cells (Figs. 4 and 5). The results suggest that DADS and TPA can inhibit the RORα/β-catenin signaling target gene through PKCα/RORα mediated by DADS and TPA, with the same effect as TPA.

Studies have shown that RORα enhances sensitivity to fluorouracil by attenuating G6PD and PFKFB3 in GC cells [7]. Study proves that PS VII can induce apoptosis of drug-resistant ovarian cancer cells by up-regulating RORα and improve drug resistance therapy [30]. NOB can overcome Adriamycin resistance in lung cancer cells, and enhance the chemotherapy to Adriamycin through the Akt/GSK3β/β-catenin/MYCN/MRP1 pathway [37]. NOB can significantly increase the chemosensitivity of ABCB1-overexpressed ovarian cancer cells and paclitaxel-resistant lung cancer cells to paclitaxel, doxorubicin, docetaxel, and daunorubicin and reverse MDR [38]. The RORα agonist can increase the apoptosis of GC SGC7901 cells, while its reverse agonist SR3335 can reduce the 5-FU-mediated apoptosis, suggesting that RORα promotes the apoptosis of human gastric cancer cells [13]. Numerous studies have shown that DADS can enhance the chemosensitivity of tumor cells through a variety of pathways [8,9]. DADS can induce chemosensitization to sorafenib, autophagy, cell cycle arrest, and inhibition of proliferation, invasion, and metastasis of hepatocellular carcinoma cells. Moreover, the combination with sorafenib is more effective [39].

This study showed that the RORα inhibitor T0901317 downregulates the expression of RORα in GC SGC7901 cells, while DADS upregulates the expression of RORα. The low expression of RORα can promote the proliferation of SGC7901 cells, up-regulate the expression of Bcl-2, P-gp, and XIAP, and down-regulate the expression of Caspase3, thus inhibiting apoptosis and reducing the chemosensitivity of cells to 5-FU. However, DADS can inhibit SGC7901 cell proliferation by up-regulating RORα, down-regulating the expression of Bcl-2, P-gp, and XIAP, up-regulating the expression of Caspase3, and increasing the apoptosis and chemosensitivity of 5-FU in SGC7901 cells (Fig. 6). The results suggest that DADS can upregulate RORα to enhance chemosensitivity.

The limitations of this study are mainly as follows. First, only the SR1078 cell line was used, which limits the generalizability and representativeness of the findings. Second, no *in vivo* validation was performed using animal models, which prevents a comprehensive assessment of the physiological relevance and translational potential of the results. Future studies should include a wider range of cell lines and incorporate animal experiments to further confirm the reliability and applicability of the findings.

## 5 Conclusion

In summary, this study further investigated DADS as a novel agonist that reduces RORα/β-catenin signaling, inhibits GC cell invasion and EMT, and enhances 5-FU chemosensitivity via PKCα/RORα mediated by SR1078 and TPA. DADS and SR1078 significantly suppressed GC cell proliferation, migration, and invasion, increased RORα expression, reduced RORα binding to β-catenin, decreased nuclear β-catenin and EMT markers, while upregulating E-cadherin. DADS and TPA upregulated PKCα/RORα signaling components, promoted PKCα-RORα interaction, and downregulated key β-catenin target genes. Additionally, DADS enhanced apoptosis and 5-FU sensitivity by regulating Bcl-2, P-gp, XIAP, and Caspase-3. These findings suggest DADS inhibits GC progression and chemoresistance through PKCα/RORα-mediated RORα/β-catenin signaling, acting similarly to SR1078 and TPA as a potential new agonist.

## Data Availability

The datasets and materials supporting the findings of this study are available from the corresponding authors upon reasonable request.

## Abbreviations

DADS: Diallyl disulfide
EMT: Epithelial-mesenchymal transition
GC: Gastric cancer
RORα: Retinoid-related orphan receptor α
PKCα: Protein kinase Cα
TPA: 12-O-tetradecanoylphorbol-13-acetate
XIAP: X-linked inhibitor of apoptosis protein
P-gp: P-glycoprotein
Bcl-2: B-cell lymphoma-2
VCR: Vincristine
5-FU: 5-Fluorouracil
